# Investigating Neurophysiological, Perceptual, and Cognitive Mechanisms in Misophonia

**DOI:** 10.3390/biology14030238

**Published:** 2025-02-26

**Authors:** Chhayakanta Patro, Emma Wasko, Prashanth Prabhu, Nirmal Kumar Srinivasan

**Affiliations:** 1 Department of Speech Language Pathology and Audiology, Towson University, Towson, MD 21252, USA; cpatro@towson.edu (C.P.);; 2 Department of Audiology, All India Institute of Speech and Hearing, Naimisham Campus, Manasagangothri, Mysore 570006, Karnataka, India; prashanthprabhu@aiishmysore.in

**Keywords:** misophonia, auditory evoked potentials, mismatch negativity, spatial release from masking

## Abstract

Misophonia is a condition characterized by intense, involuntary distress or anger in response to specific sounds, often leading to irritation or aggression. While the condition is recognized for its emotional and behavioral impacts, little is known about its physiological and perceptual effects. The current study aimed to explore the physiological correlates and perceptual consequences of misophonia through a combination of electrophysiological, perceptual, and cognitive assessments. Seventeen individuals with misophonia and sixteen control participants without the condition were compared. Participants completed a comprehensive battery of tests, including (a) cortical event-related potentials (ERPs) to assess neural responses to standard and deviant auditory stimuli, (b) the spatial release from the speech-on-speech masking (SRM) paradigm to evaluate speech segregation in background noise, and (c) the flanker task to measure selective attention and cognitive control. The results revealed that individuals with misophonia exhibited significantly smaller mean peak amplitudes of the N1 and N2 components in response to oddball tones compared to controls. This suggests a potential underlying neurobiological deficit in misophonia patients, as these components are associated with early auditory processing. However, no significant differences between each group were observed in the P1 and P2 components regarding oddball tones or in any ERP components in response to standard tones. Despite these altered neural responses, the misophonia group did not show differences in hearing thresholds, speech perception abilities, or cognitive function compared to the controls. These findings suggest that while misophonia may involve distinct neurophysiological changes, particularly in early auditory processing, it does not necessarily lead to perceptual deficits in speech perception or cognitive function.

## Simple Summary

While the emotional and behavioral effects of misophonia are well recognized, its impact on brain function and perception remains unclear. This study examined differences in brain responses to sound, speech perception in noisy environments, and cognitive abilities between individuals with and without misophonia. The results of this study suggested that individuals with misophonia exhibited reduced brain responses to unexpected sounds, indicating differences in early auditory processing. However, their hearing abilities, speech perception, and cognitive performance were comparable to those without misophonia. These results suggest that misophonia is associated with distinct neural processing differences but does not appear to affect overall auditory or cognitive function. A better understanding of these brain mechanisms may contribute to improved strategies for managing misophonia.

## 1. Introduction

Misophonia, derived from the Greek roots miso-(meaning “hatred”) and -phonia (meaning “sound”), is a condition characterized by intense negative emotional reactions—such as irritation, anger, or disgust—when individuals encounter specific “trigger” sounds [1]. Common triggers often include human-generated sounds such as chewing, slurping, sniffing, and throat clearing [2], as well as repetitive noises from objects like pen clicking or keyboard typing [3]. Beyond emotional discomfort, these reactions are often accompanied by increased autonomic arousal, leading to significant distress [4]. To manage this discomfort, individuals with misophonia may avoid situations where triggers are present or endure them with great difficulty [2]. Over time, this avoidance and distress can cause profound functional impairments, disrupting daily life and social interactions. Despite growing awareness of this condition, the underlying neurophysiological mechanisms, specific to the realm of auditory processing, and their broader perceptual consequences remain poorly understood, underscoring the need for further research.

Neurophysiological studies have begun to elucidate the underlying mechanisms of misophonia, shedding light on both cortical and functional anomalies associated with this condition. Research using fMRI has consistently highlighted hyperactivity in the anterior insular cortex (AIC; a key region in the salience network) in response to misophonic trigger sounds (e.g., [5]). This hyperactivity is accompanied by increased functional connectivity between the AIC and regions involved in emotional processing and regulation (e.g., ventromedial prefrontal cortex, posterior cingulate cortex), suggesting an exaggerated salience assigned to specific sounds in individuals with misophonia [5]. Furthermore, the anterior cingulate cortex (ACC; involved with top-down executive control) and superior temporal cortex have also shown hyperactivity, indicating a broader network dysfunction in response to misophonic stimuli [6].

On the electrophysiological front, Aryal and Prabhu [7] recently observed significant differences in the latency of P1 and N1 peaks of Auditory Late Latency Responses (ALLRs) between individuals with misophonia and controls. These latency differences suggest that individuals with misophonia may experience delayed or altered cortical processing of auditory stimuli. Similarly, Schröder et al. [8] investigated auditory processing in misophonia patients using auditory event-related potentials (ERPs) during an oddball task. They found that misophonia patients exhibited a smaller mean amplitude of the N1 peak in response to oddball tones compared to controls, with no significant differences in the P1 and P2 components. Importantly, no differences were observed in ERP components in response to standard tones, indicating that the deficits in misophonia are specific to change detection mechanisms in the auditory cortex, rather than reflecting broader auditory processing impairments.

Given this evidence, the mismatch negativity (MMN) paradigm provides a strong rationale for investigating auditory processing deficits in misophonia, as it is a well-established tool for assessing automatic change detection mechanisms in a repetitive auditory context [9]. Individuals with misophonia exhibit intense emotional responses to specific auditory triggers, and MMN, which operates without conscious attention, could reveal how these individuals’ brains respond to “deviant” sounds that deviate from the expected norm, providing insights into heightened sensitivity or aversive reactions to certain stimuli [10]. MMN is also closely tied to sensory memory processes, requiring the formation of short-term memory traces to compare incoming auditory stimuli against the established auditory context [11]. In misophonia, a diminished MMN may indicate a disrupted sensory memory system that fails to appropriately filter and encode auditory stimuli. Such deficits could cause certain sounds to be perceived as disproportionately salient, exacerbating emotional distress. In this study, MMN was utilized to investigate how these auditory processing deficits contribute to misophonia. Further, our analysis that focuses on the N1 peaks elicited by the standard and deviant stimuli, latency, and amplitude difference between the N1 peaks of the standard and deviant waveforms provides insights into early neural responses to acoustic changes. This metric helps compare findings with Schröder et al. [8], who reported that individuals with misophonia exhibit a selective reduction in N1 response to deviant stimuli, thereby diminishing the N1 amplitude difference between standard and deviant waveforms.

While the neurophysiological basis of misophonia has been increasingly studied, with some research highlighting altered cortical activity in affected individuals, the perceptual consequences of these neural changes remain largely unexplored. In particular, how individuals with misophonia process speech under adverse listening conditions—such as noisy or acoustically challenging environments—has received limited attention. Since individuals with misophonia do not typically exhibit peripheral auditory processing deficits, any difficulties in speech perception are likely to stem from central processes, particularly those involved in auditory scene analysis [12,13]. The spatial release from masking (SRM) paradigm is well suited to investigate these central mechanisms, as it examines how individuals segregate target speech from background noise based on spatial cues [14,15,16,17,18]. By comparing SRM performance in individuals with and without misophonia, this study aims to illuminate the perceptual challenges that characterize this population.

Cognitive operations, especially attention, are fundamental to the perception and processing of sensory stimuli, enabling individuals to focus on relevant information while suppressing distractions. In misophonia, the attentional process is often disrupted due to abnormally increased sensitivity to specific trigger sounds [19,20]. This increased vigilance towards aversive sounds can impede the ability to allocate attention to other important stimuli, resulting in difficulties in task performance and broader cognitive functioning. The attentional deficits observed in individuals with misophonia—such as an exaggerated focus on trigger sounds—suggest that cognitive resources are disproportionately allocated to these stimuli, thereby reducing the capacity to engage with other aspects of the environment [21]. To further investigate these attentional mechanisms in misophonia, we employed the flanker task, a controlled method for assessing selective attention. This task presents distracting stimuli (flankers) alongside a central target stimulus, requiring participants to focus on the target while inhibiting attention to the flankers [22,23]. We hypothesized that individuals with misophonia would exhibit greater difficulty disengaging attention from the flankers. This would suggest that their attention deficits are not confined to auditory stimuli alone but may extend to broader, domain-general cognitive processes.

In summary, this study aimed to investigate the neurophysiological, perceptual, and cognitive mechanisms underlying misophonia. Three tasks were utilized: ERP measurements to assess automatic change detection in auditory processing, the SRM paradigm to examine speech segregation in the presence of background noise, and the flanker task to evaluate selective attention and cognitive control. These tasks collectively offer a detailed understanding of the neural and perceptual impairments associated with misophonia.

## 2. Method

### 2.1. Participants

An a priori power analysis (effect size, *d* = 0.25, *α* = 0.05) indicated that a total sample size of 24 participants (12 per group) would be required to achieve a statistical power of 0.8. To ensure a sufficiently powered study, 35 participants (2 males, 33 females; age range: 18–30 years, *M* = 24 years, *SD* = 3 years) were recruited via word of mouth and social media. However, due to abnormally high electrode impedances during data collection, reliable ERP responses could not be recorded for two participants, leading to their exclusion from the analysis. This resulted in a final sample of 33 participants, with 16 assigned to the control group (1 male, 15 females; *M* = 24.37 years, *SD* = 1.76 years) and 17 to the misophonia group (1 male, 16 females; *M* = 23.94 years, *SD* = 2.94 years). Each participant was screened for misophonia using the Amsterdam Misophonia Scale (A-MISO-S; Schröder et al. [3]). The A-MISO-S, based on the Yale–Brown Obsessive–Compulsive Scale (Y-BOCS), includes six items assessing overall symptoms, distress, interference, effort to resist, control over symptoms, and avoidance [3]. Participants were placed in either the experimental (misophonic) group or control group based on their A-MISO-S score (0–4 subclinical, 5–9 mild, 10–14 moderate, 15–19 severe, 20–24 extreme). Scores of 5 and above indicated misophonia. Participants were blinded to the outcome of the A-MISO-S screening and were not informed of their group allocation to ensure unbiased participation in the study. Participants also described scenarios where they encountered triggers, such as in classrooms and at home, and their reactions (e.g., “anger (extreme)”, “extremely uncomfortable”, “anxiety/panic attack”, “flee the environment”, “irritated”, “distress”, “disgust”), aligning with diagnostic criteria [24,25,26,27,28]. In total, 16 participants were included in the control group (1 male, 15 females, aged 24, *M* = 24, *SD* = 2), and 19 in the misophonic group (1 male, 18 females, aged 24, *M* = 24, *SD* = 3), with an average A-MISO-S score of 9. Within the misophonic group, 11 participants scored in the mild range, 4 in the moderate range, 1 in the severe range, and 1 in the extreme range.

Participants were screened for hearing deficits through otoscopic inspection, tympanometry, and standard pure-tone audiometry. Written informed consent was obtained from all participants in accordance with the Towson University Institutional Review Board (IRB #1717, approved on 21 April 2022), Towson, MD, USA. Participants were compensated at a rate of USD 15 per hour for their time. Payment was issued in the form of gift cards upon completion of the study session.

### 2.2. ERP: Stimuli, Acquisition Parameters, and Response Analyses

ERP recordings were conducted using a pair of stimuli following an oddball paradigm (i.e., standard and deviant stimuli). The stimulus pair consisted of a 600 Hz tone presented binaurally. Each stimulus had a total duration of 200 ms, including a 30 ms rise time, a 140 ms steady-state plateau, and a 30 ms fall time. The standard stimulus was an unaltered 600 Hz tone delivered to both ears. The deviant stimulus featured the same 600 Hz tone but with a 15° phase shift (equivalent to 0.2618 radians) applied to the right channel. This phase difference corresponds to a small temporal delay relative to the left channel over the 600 Hz tone’s period (~1.67 ms). All stimuli were generated using MATLAB version 2024 (MathWorks, Inc., Natick, MA, USA).

The MMN response waveforms were obtained using a Duet two-channel SmartEP system (Intelligent Hearing Systems, Miami, FL, USA). Stimuli were presented at 70 dBnHL in an ‘oddball’ paradigm, with the standard stimulus occurring 80% of the time and the deviant stimulus 20% of the time. The stimuli were presented in rarefaction polarity, with a repetition rate of 1.1/s. Responses were averaged over 200 sweeps of deviant stimuli and 800 sweeps of standard stimuli, within a time frame of −50 to 500 ms relative to stimulus offset. A bandpass filter with a frequency range of 0.1 to 30 Hz was applied, and the signal was amplified up to 50,000 times. Stimuli were presented binaurally to each participant.

Participants were seated comfortably to minimize muscular artifacts and engaged in watching a subtitled movie without sound to ensure a passive listening state. Each participant was informed about the preparation and placement process of the electrode montage (2-channel, vertical montage). Before electrode placement, the skin was cleansed using an exfoliating gel, and an electrolytic paste was applied for electrode attachment. The electrodes were securely affixed using micropore tape in the designated regions according to the standard 10–20 electrode placement system: ground electrode (Fp), positive electrode (Fz), and reference electrodes on the mastoids (M1 for the left channel, M2 for the right channel). Foam insert earphones were placed in each participant’s ears for a binaural setup. Participants were instructed to remain quiet and focus solely on the movie content, disregarding any acoustic stimuli delivered through the earphones. Impedance levels were confirmed before each run (3–5 runs), maintaining less than 5 kΩ for absolute impedance and below 2 kΩ for inter-electrode impedance.

All ERP waveforms elicited by the standard and deviant stimuli were visually inspected, and the peak latencies and amplitudes were manually identified and marked for subsequent analysis. This manual marking ensured accuracy and consistency in capturing the electrophysiological responses across participants. The normative latency ranges for each ERP peak were referenced based on established guidelines to standardize interpretations. For the standard stimuli, the P1, N1, and P2 peaks were observed at approximate latencies of 50 ms, 100 ms, and 200 ms, respectively. These values align with expected auditory cortical responses to simple auditory stimuli under normal conditions [28]. For the deviant stimuli, the same peaks typically appeared within similar latency ranges, although slight shifts in latency and amplitude were occasionally observed, likely reflecting stimulus-specific neural processing.

To isolate components related to auditory deviance detection, difference waveforms were calculated by subtracting the ERP elicited by standard stimuli from that elicited by deviant stimuli for each trial. Subsequent analyses focused on two primary negative peaks: ΔN1 and MMN. ΔN1 was defined as the difference in latency and amplitude between the N1 peaks of the standard and deviant waveforms, typically occurring within the 80–120 ms latency range. In contrast, MMN was identified as a distinct negative deflection in the difference waveform, occurring within the 150–250 ms latency range. MMN amplitude was calculated as the difference between the N2 peak elicited by deviant stimuli and the corresponding segment of the standard waveform [9]. This measure reflects neural processes involved in the automatic detection of auditory irregularities. In addition to amplitude measures, we analyzed the latencies of individual ERP components and difference waveforms. The latency analyses provided complementary insights into the temporal dynamics of neural responses [29], offering a detailed perspective on the temporal dynamics of auditory processing and potential deficits associated with misophonia.

### 2.3. SRM Paradigm

The speech corpus recorded at Boston University’s Hearing Research Center [15] was used to assess SRM. This corpus contained sentences composed of five monosyllabic words that are syntactically correct but semantically unpredictable. Prior research has shown that access to semantic cues may obscure the observation of any potential relationship between misophonia and speech perception. The target (and the masker) sentences in this corpus had the structure <name> <verb> <number> <adjective> <object> (e.g., Jane found five red toys), with each word being randomly selected from 8 possible options. The target always contained the <name> call-sign “Jane” with other keywords being randomly selected from other available choices. The target was always presented in the center (0° azimuth), while the maskers were either colocated with the target at 0° azimuth or spatialized at ±15° (spatially separated). The spatial locations of the target and background speakers were simulated by applying non-individualized head-related transfer functions (HRTFs) to the test stimuli, downloaded from an MIT website (sound.media.mit.edu). The target/masker voices (female voice: f0 = 175–200 Hz) varied across trials but remained the same within each given trial. The stimuli were generated in MATLAB version 2024 (MathWorks, Inc., Natick, MA, USA) on a PC and played using a Lynx Hilo sound card (Lynx Studio Technology, Costa Mesa, CA, USA). The stimuli were presented via Sennheiser HDA 650 (Sennheiser, Old Lyme, CT, USA) headphones. The masking stimuli were fixed at 70 dB SPL, while the target signal level was adaptively adjusted using a one-up, one-down tracking procedure to determine the Target-to-Masker Ratio (TMR) threshold corresponding to 50% correct performance. SRM was quantified by subtracting the TMR threshold of the spatially separated condition from that of the colocated condition, representing the improvement in speech perception due to spatial separation. The methodology for stimulus presentation and response analysis for SRM has been detailed in recent studies conducted by our laboratory [30,31,32,33]. Participants were tasked with identifying target sentences beginning with the call-sign “Jane”, always delivered from the center. Each trial featured a randomized pairing of target and masker voices, consistent within a given trial. Practice runs for both conditions were conducted to familiarize participants with the task. The experiment used a fixed set of conditions, and the target–masker configurations were randomized to minimize predictability effects.

### 2.4. Flanker Task

The flanker test was conducted using the Psychology Experiment Building Language (PEBL version 2.0.4) platform (The PEBL Project, Fairfax, VA, USA) [34]. In this task, participants were presented with a central stimulus and tasked with identifying its left–right orientation while actively disregarding surrounding stimuli, referred to as ‘flankers’ [35]. The primary objective was to determine whether the centrally located arrow pointed to the left or right, while ignoring the orientation of the surrounding arrows [36]. The surrounding arrows were either congruous, where the target stimulus and distracting stimuli were associated with the same response (e.g., all arrows pointing in the same direction), or incongruous, where the target stimulus and distracting stimuli were associated with different responses (e.g., the central arrow pointing left while flankers point right) [37]. The key manipulation in this task was the congruency of the target stimulus and its flankers.

Each participant underwent 160 trials, with accuracy and response times recorded. These measurements served as indicators of cognitive attention, in line with the methodology detailed by Zelazo et al. [38]. The task setup is straightforward, with stimuli shown for an unlimited time until the participant responds to advance to the next trial. The stimuli were typically composed of a target image in the center flanked by one or more distractor stimuli on either side. Completion times for the flanker task were scored separately for congruent, incongruent, and conflict cost conditions. To account for potential confounding factors such as motor skills, visual acuity, and processing speed, a differential measure termed conflict cost was employed. Conflict cost was calculated by subtracting the total completion times for congruent trials from those for incongruent trials, providing a more targeted assessment of attentional control [35]. Higher conflict costs would indicate greater difficulty in filtering out irrelevant distractors and focusing on the target stimulus, suggesting weaker attentional control. Conversely, lower conflict costs demonstrate an enhanced ability to manage interference and maintain focus. By analyzing conflict cost, this study aimed to gain insight into the attentional processes that may be disrupted in individuals with misophonia.

### 2.5. Testing Procedure

The participants’ testing journey began with audiometric assessments to establish baseline hearing thresholds. Following this, the A-MISO-S scale was used to assess the severity of misophonia symptoms, providing context for individual differences in misophonia severity. Next, participants completed the ERP task to examine neural responses to auditory deviance, focusing on MMN and ΔN1 components. This task assessed the brain’s automatic response to standard and deviant auditory stimuli, providing insights into auditory processing and potential neural impairments associated with misophonia. The SRM paradigm followed, where participants’ ability to segregate speech from background noise was tested. By measuring SRM, this task evaluated how well participants could perceive speech in noisy environments, highlighting potential challenges faced by individuals with misophonia in real-world listening situations. Finally, participants engaged in the flanker task, which assessed cognitive control and attentional processes. The conflict cost measure was used to examine participants’ ability to filter distractions and focus on the target stimulus, offering insights into the attentional challenges that may be present in individuals with misophonia.

## 3. Results

### 3.1. Audiometric Characteristics and Misophonia Severity

**Figure 1:**
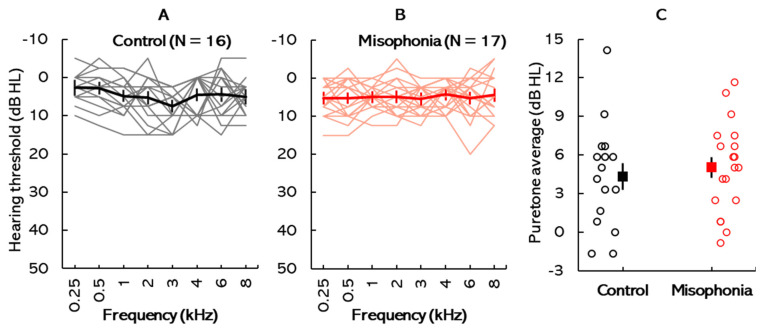
Audiometric characteristics: (**A**) Individual and mean audiograms (averaged across ears) for the control group (**A**) and misophonia group (**B**). Thin colored lines represent individual thresholds, while bold lines indicate the group mean thresholds. (**C**) Comparison of individual and mean pure-tone average thresholds between the control and misophonia groups.

All participants enrolled in the study exhibited symmetrical hearing abilities bilaterally, with interaural asymmetries of 10 dB HL or less across all tested frequencies. Figure 1 illustrates the audiometric thresholds for both the control and misophonia groups. Figure 1A,B display individual audiograms, along with mean audiograms averaged across ears, for the control group and the misophonia group, respectively. Thin colored lines represent the hearing thresholds of individual participants, while bold lines highlight the group mean thresholds. Notably, all participants displayed thresholds within the normative range (≤20 dB HL). Figure 1C compares the individual and mean PTA thresholds between the two groups. A mixed-design analysis of variance (ANOVA) was conducted to compare auditory thresholds between the two test groups. Test frequency served as the within-subject factor, while subject group served as the between-subject factor. The results revealed a significant main effect of test frequency [*F*(7, 217) = 1.01, *p* = 0.04, *η*^2^ = 0.03]. However, the main effects of participant group [*F*(1, 31) = 0.27, *p* = 0.61, *η*^2^ = 0.01] and its interaction with test frequency [*F*(7, 217) = 2.01, *p* = 0.60, *η*^2^ = 0.06] were not statistically significant. Post hoc pairwise comparisons indicated that intergroup differences were not statistically significant at any of the test frequencies (all *p* > 0.05). These findings suggest similar audiometric thresholds across all tested frequencies for both participant groups. Additionally, the pure-tone averages (PTAs) were calculated for each participant and compared between the control (M = 4.33, SD = 3.44) and misophonia (M = 5.5.04, SD = 3.01) groups using an independent-sample *t*-test. The results showed no significant difference in PTA between the two groups (*t*(31) = −0.39, *p* = 0.70, *Cohen’s d* = 0.08). The mean PTAs are depicted in Figure 1C.

**Figure 2:**
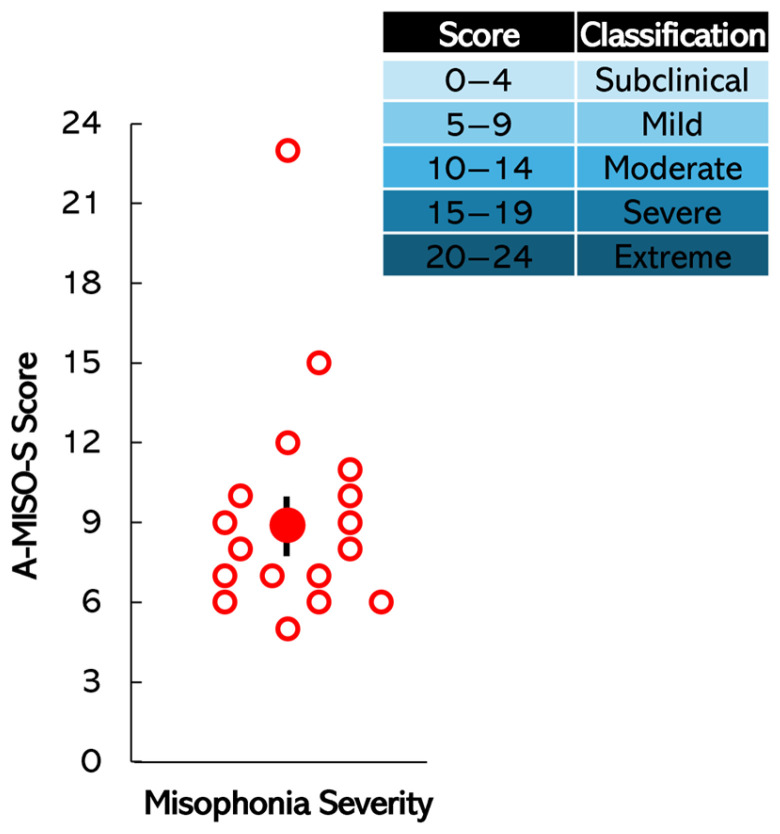
Misophonia severity: Individual data distribution (unfilled circles) and group mean A-MISO-S score (filled circle) are shown, highlighting the variability within the sample and the average severity. The error bars indicate ±1 standard error (SE). The degree of misophonia severity, as defined by Schröder et al. [8], is also included for reference.

The distribution of individual A-MISO-S scores (unfilled circles) and the group mean score (filled circle) are presented in Figure 2, highlighting both the variability within the sample and the average severity of misophonia. Error bars represent ±1 standard error (SE). For reference, the degree of misophonia severity, as defined by Schröder et al. [8], is also indicated, providing context for interpreting the severity levels within the sample. This visualization underscores the range of experiences among participants while situating the group mean within established severity thresholds. It is evident from the figure that all participants in the misophonia group exhibited clinically significant misophonia, as indicated by their total A-MISO-S scores falling within the range of 5 to 24. The mean A-MISO-S score among these participants was calculated at 8.89, demonstrating a considerable level of symptom severity across the sample, with individual scores ranging from 5 to 23.

### 3.2. ERP Amplitudes and Latencies

**Figure 3:**
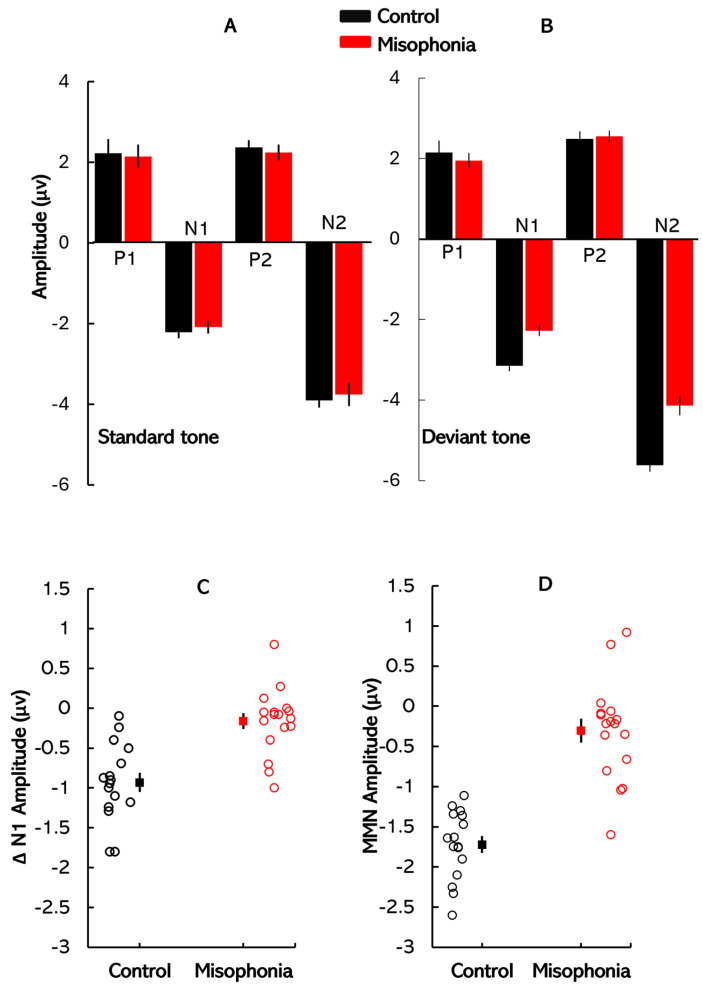
ERP amplitude: Mean ERP amplitudes for the control and misophonia groups obtained using (**A**) standard stimuli and (**B**) deviant stimuli. Groups are color-coded for clarity, with the control group in black and the misophonia group in red. (**C**) Individual data distribution (unfilled circles) and mean ΔN1 amplitudes, and (**D**) individual data distribution and mean MMN amplitudes for the misophonia and control groups. Error bars in all figures represent ±1 SE.

Figure 3 displays the mean amplitudes of the ERP components (P1, N1, P2, and N2) for the control and misophonia groups across different stimulus conditions. Panels A and B show the mean amplitudes elicited by standard stimuli and deviant stimuli, respectively, with groups color-coded for clarity (green for the control group and purple for the misophonia group). Panels C and D present the individual data distribution (unfilled circles) and mean amplitudes for ΔN1 and MMN responses, respectively, for both groups. Error bars in all panels represent ±1 standard error (SE). Two mixed-design ANOVAs were conducted to analyze these amplitudes: one for responses to standard tones and another for responses to deviant tones across the two groups. In each ANOVA, ERP component (P1, N1, P2, N2) served as the within-subject factor, and group (control vs. misophonia) served as the between-subject factor. For the standard tones, there was a significant main effect of ERP component [*F*(3, 93) = 585.74, *p* < 0.001, *η*^2^ = 0.95], indicating that cortical response amplitudes differed between the four peaks. However, neither the main effect of group [*F*(1, 31) = 0.04, *p* = 0.85, *η*^2^ < 0.001] nor the interaction between ERP component and group [*F*(3, 93) = 0.11, *p* = 0.95, *η*^2^ < 0.001] reached statistical significance. In contrast, analysis of responses to the deviant tones revealed a significant main effect of ERP component [*F*(3, 93) = 895.73, *p* < 0.01, *η*^2^ = 0.97], suggesting distinct cortical responses to deviant stimuli. While the main effect of group was not significant [*F*(1, 31) = 9.88, *p* < 0.01, *η*^2^ = 0.24], a significant interaction between ERP component and group was observed [*F*(3, 93) = 5.58, *p* < 0.01, *η*^2^ = 0.15]. Post hoc pairwise comparisons were conducted to examine this interaction. No significant group differences were found in P1 (*Mdiff* = 0.24, *SE* = 0.35, *p* = 0.49, *Cohens’ d* = 0.10) or P2 amplitudes (*Mdiff* = 0.01, *SE* = 0.23, *p* = 0.99, *Cohen’s d* = 0.01). However, significant group differences were observed for N1 (*Mdiff* = −0.86, *SE* = 0.19, *p* < 0.01, *Cohen’s d* = −1.24) and N2 amplitudes (*Mdiff* = −1.55, *SE* = 0.29, *p* < 0.01, *Cohen’s d* = −1.27), with the misophonia group showing reduced amplitudes compared to the control group.

To further examine group differences, ΔN1 and MMN waveforms were calculated by subtracting the ERP waveforms elicited by standard tones from those elicited by deviant tones. Two independent *t*-tests were performed to compare the ΔN1 and MMN response amplitudes between the control and misophonia groups. The first *t*-test revealed a significant reduction in ΔN1 amplitudes in the misophonia group compared to the control group (*t*(31) = −4.90, *p* < 0.01, *Cohen’s d* = −1.52). Similarly, the second *t*-test demonstrated a significant reduction in MMN amplitudes in the misophonia group (*t*(31) = −7.63, *p* < 0.01, *Cohen’s d* = −2.38). These findings suggest that both ΔN1 and MMN response amplitudes were significantly diminished in individuals with misophonia compared to controls. The mean ΔN1 and MMN amplitudes for each group are depicted in Figure 3B and Figure 3C, respectively.

**Figure 4:**
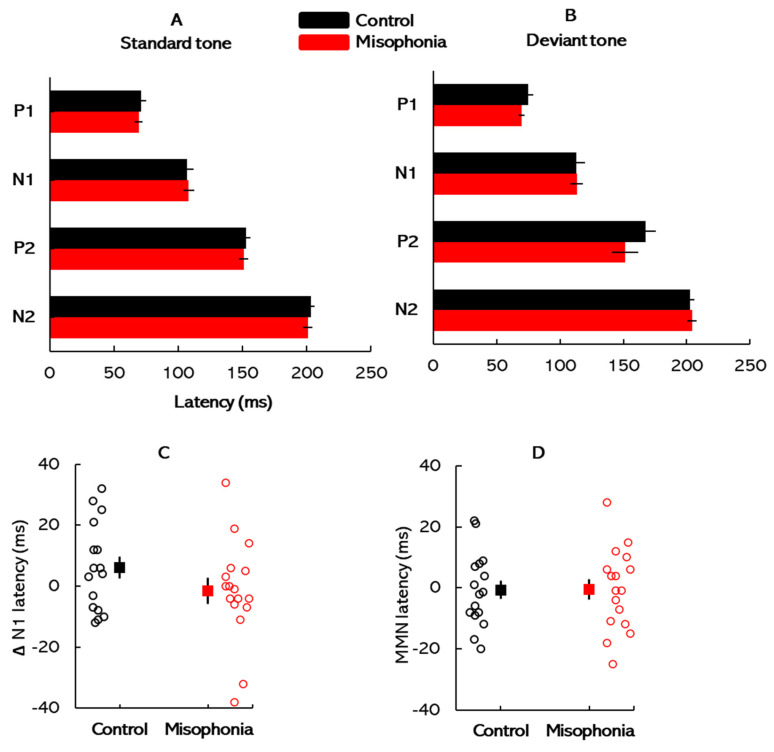
ERP latency: Mean ERP latencies for the control and misophonia groups obtained using (**A**) standard stimuli and (**B**) deviant stimuli. (**C**) Individual data distribution (unfilled circles) and mean ΔN1 latencies, and (**D**) individual data distribution and mean MMN latencies for the misophonia and control groups. Error bars in all figures represent ±1 SE.

Figure 4 shows the mean ERP latencies for the control and misophonia groups across stimulus conditions. Panels A and B present the latencies elicited by standard and deviant stimuli, respectively. Panels C and D illustrate the individual data distribution (unfilled circles) and mean latencies for ΔN1 and MMN responses, respectively, in both groups. Aligned with the ERP amplitude analyses, we conducted two separate mixed-design ANOVAs to compare ERP peak latencies across participant groups: one for standard tones and another for deviant tones. For standard tones, the analysis revealed a significant main effect of ERP component [*F*(3, 96) = 1126.14, *p* < 0.001, *η*^2^ = 0.97], indicating that response latencies varied significantly across the different ERP components. However, neither the main effect of group [*F*(1, 31) = 0.05, *p* = 0.82, *η*^2^ < 0.001] nor the interaction between ERP component and group [*F*(3, 93) = 0.44, *p* = 0.72, *η*^2^ = 0.01] was statistically significant. Similarly, for deviant tones, the analysis revealed a significant main effect of ERP component [*F*(3, 93) = 227.16, *p* < 0.01, *η*^2^ = 0.88). However, neither the main effect of group [*F*(1, 31) = 1.32, *p* = 0.26, *η*^2^ = 0.04] nor the interaction between ERP component and group [*F*(3, 93) = 0.97, *p* = 0.97, *η*^2^ < 0.001] was significant. Further, independent *t*-tests were conducted to compare the latencies of the ΔN1 and MMN components between groups. The *t*-test comparing ΔN1 latency revealed no significant difference between the groups (*t*(31) = 0.38, *p* = 0.35, *Cohen’s d* = 0.12). Similarly, the *t*-test comparing MMN latency also showed no significant group difference (*t*(31) = −0.04, *p* = 0.48, *Cohen’s d* = −0.01). The latencies of the ΔN1 and MMN components for each group are depicted in Figure 4C and Figure 4D, respectively.

### 3.3. SRM

**Figure 5:**
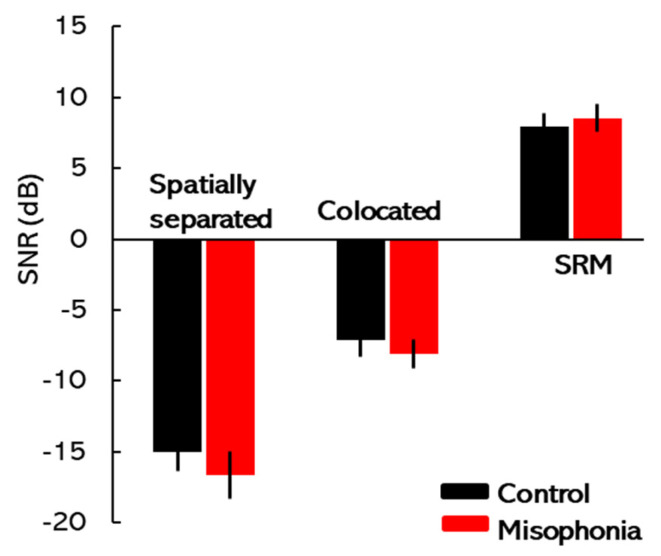
Spatial release from masking (SRM): Mean TMRs are plotted for colocated conditions (target and maskers at 0° azimuth) and spatially separated conditions (target at 0° and maskers at ±15° azimuths) for the control and misophonia groups. The amount of SRM for both groups is also presented. Error bars represent ±1 SE.

Figure 5 depicts the mean Target-to-Masker Ratios (TMRs) for colocated conditions (target and maskers at 0° azimuth) and spatially separated conditions (target at 0° and maskers at ±15° azimuths) in the control and misophonia groups. The magnitude of spatial release from masking (SRM) for each group is also presented. The results of the two-way mixed-design ANOVA, which included masker spatial location as the within-subjects factor and participant group as the between-subject factor, revealed a significant main effect of masker spatial location [*F*(1, 31) = 154.25, *p* < 0.01, *η*^2^ = 0.83]. This indicates that SNR thresholds varied significantly between colocated and spatially separated conditions. However, the main effects of participant group [*F*(1, 31) = 0.20, *p* = 0.66, *η*^2^ = 0.01] and the interaction between participant group and masker spatial location [*F*(1, 31) = 0.20, *p* = 0.66, *η*^2^ = 0.01] were not statistically significant.

Post hoc pairwise comparisons for the within-subject factor confirmed that both groups exhibited a significant benefit from target–masker spatial separation, showing substantially better speech recognition performance in the spatially separated condition compared to the colocated condition (*p* < 0.01). Conversely, between-group comparisons revealed no significant differences in speech recognition performance between the control and misophonia groups under either spatial condition (spatially separated: *p* = 0.34; colocated: *p* = 0.51). To compare the extent of spatial release from masking (SRM) between the groups, SRM values were calculated for each participant by subtracting the TMR thresholds in the colocated condition from those in the spatially separated condition. An independent-sample *t*-test revealed no significant difference in SRM scores between the groups (*t*(31) = −0.45, *p* = 0.33, *Cohen’s d* = −0.16), indicating that both groups benefitted equally from spatial separation of the target and masker (see Figure 5).

### 3.4. Flanker Task

**Figure 6:**
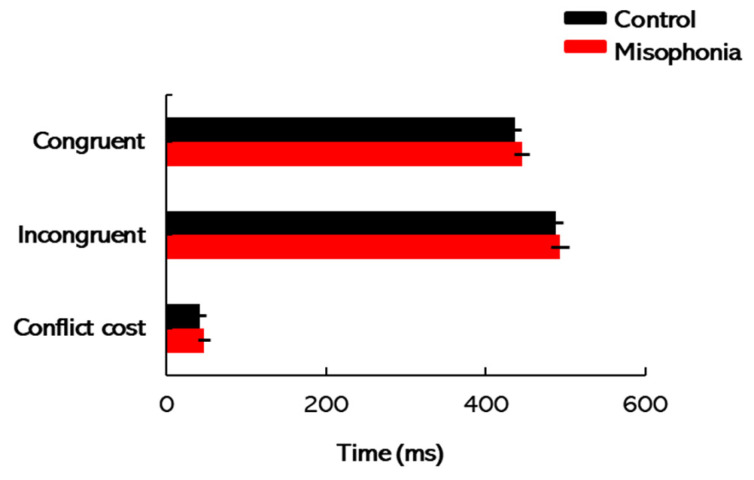
Flanker task: Mean completion times for the congruent and incongruent sections of the flanker task, along with the conflict cost (the difference in completion times between these two conditions), are presented. Error bars represent ±1 SE.

Figure 6 illustrates the mean completion times for the congruent and incongruent sections of the flanker task, along with the conflict cost, defined as the difference in completion times between these two conditions. A two-way mixed-design ANOVA was conducted with task type (congruent vs. incongruent) as the within-subject factor and participant group as the between-subject factor. The analysis revealed a significant main effect of task type [*F*(1, 31) = 225.05, *p* < 0.01, *η*^2^ = 0.88], indicating that completion times were significantly longer for incongruent trials compared to congruent trials. However, neither the main effect of group [*F*(1, 31) = 2.32, *p* = 0.19, *η*^2^ = 0.07] nor the interaction between task type and group [*F*(1, 31) = 0.80, *p* = 0.40, *η*^2^ = 0.02] was significant. These results suggest that both groups exhibited similar flanker completion times across the task conditions. The analysis of conflict cost also revealed no significant difference between the control and misophonia groups [*t*(31) = −0.89, *p* = 0.38, *Cohen’s d* = −0.32]. These findings indicate that both groups exhibited comparable abilities to focus attention and suppress interference from distracting stimuli, as measured by the flanker task. Overall, the results suggest that misophonia is not associated with deficits in selective attention or cognitive control.

### 3.5. Correlation Analyses

**Figure 7:**
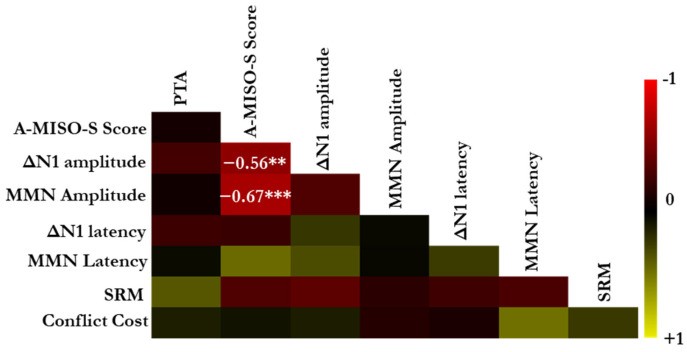
Multiple correlations: A heat map illustrating the strength of associations between the various measures used in this study. Significant correlations are denoted by asterisks (*** *p* < 0.001 and ** *p* < 0.01) at the uncorrected significance level for multiple comparisons. Nonsignificant correlations are not marked with asterisks.

Data from the 17 participants with misophonia were analyzed to explore the associations between the obtained measures using multiple correlations. Figure 7 presents a heat map illustrating the strength of correlations between the various measures used in this study. Significant correlations are denoted by asterisks (*** *p* < 0.001 and ** *p* < 0.01), based on the uncorrected significance level for multiple comparisons. Nonsignificant correlations are not marked. This visualization provides an overview of the relationships between behavioral, neural, and self-report measures, highlighting key associations relevant to understanding misophonia. A Bonferroni-adjusted significance level (α = 0.002) was applied to account for the 28 comparisons. Two significant correlations were observed. The A-MISO-S scores, which reflect self-reported estimates of misophonia severity, showed a significant negative correlation with both ΔN1 amplitude (*r* = −0.56, *p* = 0.019) and MMN amplitude (*r* = −0.67, *p* < 0.002). These negative correlations indicate that as misophonia severity increases, the amplitudes of these ERP components tend to decrease. However, only the correlation between A-MISO-S scores and MMN amplitude remained significant after applying the adjusted alpha threshold (*α* = 0.002), indicating that the relationship between misophonia severity and cortical response is particularly evident in the MMN component. All other correlations were not significant, even at the uncorrected alpha level (all *p* > 0.05). Only noteworthy (lack of) correlations are highlighted below.

One of the hypotheses of this study was to assess whether individuals with misophonia exhibit speech perception deficits. The group comparison results indicated no significant difference in SRM between the groups. Notably, no correlation was also found between SRM and A-MISO-S scores, suggesting that misophonia severity was not associated with speech perception in noise. Furthermore, ERP components were not significantly correlated with SRM, indicating that the physiological deficits observed at the cortical level in individuals with misophonia, as reflected by the ERP measures, did not necessarily translate into perceptual deficits in speech perception.

Additionally, no correlation was found between A-MISO-S scores and either ΔN1 latency or MMN latency. Although the amplitudes of these ERP components were reduced in individuals with misophonia, the latencies were not significantly altered, suggesting that misophonia severity was not associated with changes in the timing of neural responses.

Finally, the PTAs were not correlated with any of the measures used in the study. More importantly, neither ΔN1 amplitudes nor MMN amplitudes were correlated with PTA values, suggesting that the observed individual differences in cortical responses were not driven by differences in audibility. Instead, these differences appear to reflect variations in misophonia severity.

## 4. Discussion

While significant progress has been made in recognizing and establishing diagnostic criteria for the condition, the underlying pathophysiological mechanisms driving misophonia remain elusive. One area of conflict is understanding how the brain processes auditory stimuli in individuals with misophonia compared to those without the condition. The objective of this study was to delve deeply into the various facets of physiological, perceptual, and cognitive processing in individuals exhibiting misophonia symptoms, contrasting them with those who do not manifest such symptoms.

### 4.1. Hearing Sensitivity Is Unaffected by Misophonia

In this study, no findings consistent with individuals with misophonia having hearing loss were observed. Both individual and group audiograms revealed that pure-tone averages did not differ significantly between individuals with and without misophonia. This aligns with previous research indicating that no significant hearing loss is typically observed in individuals with misophonia. Other studies have similarly found no substantial evidence of hearing disorders among individuals with misophonia [3,4,39]. For instance, Jager et al. [4] found that hearing loss prevalence in individuals with misophonia was lower than in the general population, suggesting misophonia is not closely linked to hearing loss. Similarly, Siepsiak et al. [39] reported no significant differences in pure-tone thresholds or loudness discomfort levels between individuals with misophonia and healthy controls, further supporting the absence of a direct association between misophonia and hearing ability. Aryal and Prabhu [7] also compared hearing thresholds in individuals with and without misophonia across standard (0.25–8 kHz) and extended high frequencies (9–16 kHz). Their results showed similar hearing abilities in both groups, indicating that misophonia may not affect auditory sensitivity. Thus, the findings of this study are consistent with this body of research and contribute to the growing literature supporting the absence of significant hearing loss in individuals with misophonia.

### 4.2. Misophonia Severity: Consistency with Existing Research

The average severity of misophonia symptoms in our study participants was 8.89 on the A-MISO-S scale, which falls within the mild range, consistent with Schröder et al. [3]. Similar findings have been reported in previous studies, where 35% to 50% of individuals in help-seeking or student samples exhibited mild symptoms. For example, Naylor et al. [40] found that clinically significant misophonic symptoms were common, affecting 49.1% of their sample in the U.K. Within this experimental group, 37% of participants experienced mild symptoms, 12% had moderate symptoms, and 0.3% had severe symptoms, with no extreme cases identified. Our study’s findings are consistent with Aryal et al. [7], who researched the Indian population and found that the majority of participants with misophonia exhibited mild symptoms (22.35%). The A-MISO-S questionnaire demonstrated good internal consistency and validity for assessing misophonia symptoms among the student population at Towson University. These results suggest that while a significant number of individuals experience mild symptoms, only a small subset exhibit severe symptoms. Overall, the severity levels observed in our study align with those reported in previous population-based research, highlighting the representative nature of our misophonia sample.

### 4.3. Physiological Correlates of Misophonia

The most significant finding of this study was the significantly reduced mean amplitude of the auditory N1 and N2 waves in individuals with misophonia compared to the control group. This reduction in N1 amplitude was observed solely in response to deviant stimuli, with no significant differences for standard stimuli. Consequently, the derived ΔN1 and MMN response amplitudes, calculated from the N1 amplitudes, were also significantly diminished in the misophonia group.

These findings suggest that misophonia may involve irregular processing of specific auditory stimuli, particularly those perceived as deviant or unexpected. Our study aligns with Schröder et al. [8], who also observed a reduction in the N1 amplitude in response to oddball tones in individuals with misophonia. In their study, regularly repeated 1000 Hz tones were interspersed with deviant 250 Hz and 4000 Hz tones. Similarly, we found no significant differences in the P1 and P2 components elicited by oddball tones, and the latencies of these ERP components were unaffected, mirroring Schröder et al.’s [8] findings. Furthermore, our study provides additional evidence that, in individuals with misophonia, not only is the N1 amplitude diminished in response to deviant tones, but the N2 component is also reduced. This further supports the notion that misophonia may involve altered neural processing of unexpected auditory stimuli.

Schröder et al. [8] proposed two possible explanations for the reduced N1 amplitude observed in individuals with misophonia: increased mood disturbance and psychiatric comorbidities. Misophonia patients in their study reported significantly higher mood disturbance scores, suggesting a state of general hyperarousal. This heightened arousal could potentially diminish attention to sounds, explaining the reduction in N1 amplitude. They also considered the influence of psychiatric comorbidities and psychotropic medication, factors not assessed in our study, which may have impacted the N1 response (and possibly N2) in the misophonia group. The N1 component is crucial in studies of auditory attention, particularly in disorders like schizophrenia, where attenuated N1 responses have been frequently observed. N1 and N2 is believed to serve as a marker for early attention to sensory changes [41] and may help identify significant auditory events [42]. However, our study found no behavioral evidence of attention deficits in misophonia participants, as assessed by the flanker task. This suggests that attentional impairments in misophonia, if present, might be too subtle to detect through behavioral measures.

Additionally, since the flanker task primarily assesses visual attention, it may not be sensitive enough to detect auditory attention deficits specific to misophonia. A task designed to assess auditory attention would likely provide better insight into the attentional mechanisms involved in misophonia. Regarding ERP component latencies, no significant group differences were observed, consistent with Schröder et al. [8]. These findings suggest that misophonia may not be associated with alterations in the timing of neural responses as measured by ERP components. However, Aryal and Prabhu [43] reported shortened latencies of early ERP components, particularly P1 and N1, in misophonia patients, suggesting increased activity in subcortical auditory structures. Our study did not observe this shortening, which may be attributed to methodological differences. Specifically, while we used an oddball paradigm, Aryal and Prabhu [43] employed a standard 500 Hz tone burst stimulus, which could explain the discrepancy in latency findings.

In summary, our findings contribute to the growing body of research on misophonia’s neurophysiological mechanisms, particularly its impact on the processing of deviant auditory stimuli. Further research is needed to explore the potential role of subcortical auditory structures and refine our understanding of how misophonia affects attention and sensory processing.

### 4.4. No Evidence of Perceptual Deficits in Misophonia

In our study, speech perception abilities were similar across participant groups, and misophonia was not found to be associated with deficits in speech perception in noise. This lack of association could be explained by two possibilities. First, the altered cortical functions associated with misophonia may not significantly impact the ability to perceive speech in noisy environments. This would suggest that the neural mechanisms involved in misophonia are separate from those involved in speech processing in noise. Second, the speech task used in this study may not have been sensitive enough to detect more subtle perceptual deficits in individuals with misophonia. The SRM task might not have adequately challenged participants or captured the specific auditory processing issues that individuals with misophonia experience. To our knowledge, no study has systematically evaluated the effect of misophonia on speech perception. This underscores the need for further research to explore the potential impact of misophonia on speech perception abilities, particularly under varied listening conditions. A conference proceeding by investigated speech perception in noise among individuals with misophonia and reported deficits in noisy environments [44]. However, detailed methodological information (e.g., stimuli used, severity of misophonia, comorbid status of the participants) is lacking, making it challenging to directly compare their findings with ours. These discrepancies in methodological parameters across studies could contribute to differences in outcomes regarding the relationship between misophonia and speech perception abilities. Therefore, further research with well-defined methodologies and comprehensive assessments is needed to better understand the impact of misophonia on speech perception.

## 5. Limitations

While this study offers valuable insights into the potential impact of misophonia on auditory function, it is important to acknowledge several limitations that could affect the interpretation of the findings. Firstly, the relatively small sample size of 33 participants (17 misophonics, 16 control subjects) raises concerns about the generalizability of the results. A larger and more diverse sample could enhance the statistical power of the study, allowing for more robust conclusions and better detection of subtle differences between groups. Secondly, the study exclusively focused on college students, which may not accurately represent the broader population affected by misophonia. Misophonia can affect individuals of all ages and backgrounds, and limiting the study to this demographic may restrict the applicability of the findings to other age groups or populations. Furthermore, the study did not account for potential comorbid conditions that could influence physiological measurements. Misophonia often coexists with other psychiatric or neurological disorders, such as anxiety or obsessive–compulsive disorder, which could confound the results. Future research should consider controlling these comorbidities to better isolate the effects of misophonia on auditory function. Similarly, another limitation of this study is that we neither asked participants nor explicitly screened for the presence of tinnitus or hyperacusis. As these conditions could influence auditory cortical potentials and confound the findings in the misophonia group, this should be considered when interpreting the results. Future studies should account for these factors to more accurately isolate the neural and perceptual effects of misophonia.

Another key limitation of the present study is that cognitive status was assessed solely with the flanker task, which, while informative, does not provide a comprehensive evaluation of overall cognitive function. Therefore, conclusions about participants’ general cognitive status cannot be made based on this test alone. Similarly, ERP analysis focused only on cortical responses to standard and deviant tones, specifically the P1-N1-P2-N2 components. This limits the scope of the findings, as it does not include the brainstem or other auditory and cognitive potentials that may provide a fuller picture of misophonia-related neural processes. Future research should include a wider range of neurophysiological measures for a more complete understanding of the neural mechanisms involved in misophonia.

Lastly, the use of the A-MISO-S questionnaire as the primary measure of misophonia severity relies on self-reporting, which introduces the possibility of response bias or inaccurate reporting. Individuals may under-report or over-report their symptoms, leading to potential inaccuracies in the assessment of misophonia severity. Supplementing self-report measures with objective measures of misophonia, such as physiological assessments or behavioral observations, could provide a more comprehensive understanding of the condition. Future research with larger and more diverse samples, consideration of comorbid conditions, and incorporation of objective measures of misophonia could further advance our understanding of this complex disorder.

## 6. Conclusions

The results of this study suggest selective deficits in cortical event-related ERPs in individuals with misophonia, specifically reduced amplitudes of the N1 and N2 components in response to deviant stimuli, compared to the control group. While these findings provide an electrophysiological basis for misophonia, no perceptual consequences were observed, particularly in terms of speech perception or cognitive function. Despite the altered cortical processing associated with misophonia, participants did not exhibit significant differences in hearing thresholds, speech perception abilities, or cognitive function compared to controls. These results indicate that while misophonia may involve distinct neurophysiological changes, it does not necessarily lead to perceptual deficits in speech perception or cognitive function, suggesting that the disorder primarily affects neural processing without significantly impacting perceptual outcomes.

## Data Availability

Data are contained within the article.
